# Self-Transcendence in Mountaineering and BASE Jumping

**DOI:** 10.3389/fpsyg.2018.02686

**Published:** 2019-01-08

**Authors:** Erik Monasterio, C. Robert Cloninger

**Affiliations:** ^1^Canterbury District Health Board Regional Forensic Service, Christchurch, New Zealand; ^2^Psychological Medicine, Christchurch School of Medicine, University of Otago, Christchurch, New Zealand; ^3^Department of Psychiatry, Washington University in St. Louis, St. Louis, MO, United States

**Keywords:** self-transcendence, mountaineering, BASE jumping, personality, temperament, character, elite performance

## Abstract

The “extreme sports” of mountaineering and BASE Jumping are growing in popularity and are associated with significant risk of injury and death. In recent years there have also been increasing numbers of reports of reckless disregard and selfishness in the pursuit of mountaineering goals, including severe environmental degradation. Extant research has focused predominantly on personality variables that contribute to engagement, participation, and stress responsivity in these extreme sports. The Temperament and Character Inventory (TCI) provides a comprehensive account of personality traits, measuring seven dimensions of personality that are moderately heritable and associated with distinct brain networks and psychological characteristics. One of these traits is Self-Transcendence, which is associated with spiritual ideas and experiences, such as searching for something elevated and greater than one's individual self. High Self-Transcendence can motivate people to act altruistically even if that requires personal sacrifices and hardship. This article draws on the extant research literature, which has consistently found that despite substantial heterogeneity in their individual personality profiles, mountaineers, and BASE jumpers are adventurous in temperament and highly self-controlled and organized in character. Between 75 and 85% of the character configurations observed in these populations are associated with low Self-Transcendence. The purpose of this paper is to consider the role of Self-Transcendence and its effect on individual personality profiles of extreme athletes, in particular in moderating potentially self- destructive, and regressive ethical and moral behaviors in mountaineering and BASE jumping.

## Introduction

“Extreme sports” are a diverse group of sporting activities, including mountaineering and BASE Jumping, which require very high levels of skill and are physically and mentally demanding. They are associated with high risk of injury and death (Monasterio, 2005; Mei-Dan et al., 2012, 2013). Paradoxically despite these well-documented risks there has been a rapid growth in interest and participation in these sports, far more so than traditional sports, over the past 20 years (Pain and Pain, 2005).

Arguably mountaineering is the extreme sport that has been practiced the longest. Traditional mountaineering values were shaped around the concept of “the brotherhood of the rope,” which emphasized the values of fellowship, mutual support and self-sacrifice. For example, in 1953 during an attempt to be the first to climb K2, the notoriously dangerous second highest peak in the world, Dr. Charlie Houston and 6 teammates battled exhaustion, atrocious weather and frostbite to rescue a critically sick companion, Art Gilkey, from the upper reaches of the mountain. They were within reach of the summit but instead they dedicated all their energy to a harrowing rescue effort. They repeatedly put their lives at risk and survived arguably the most famous fall in mountaineering history after an ice ax belay from one of the party prevented them all from plummeting down a steep slope to their deaths. Gilkey was subsequently lost in what was assumed to be an avalanche, although Dr. Houston believed Gilkey ended his own life by cutting safety lines attaching him to the mountain to spare further risk to the team (Martin and Charles, 2009).

In August 2008 during the worst single accident in the history of K2 mountaineering a solo Sherpa, Pemba Gyalje, took enormous risks as he repeatedly climbed back up the mountain to search and rescue injured climbers. Before launching on the rescue missions Pemba had already climbed the mountain and survived a difficult and traumatic descent through the night after an ice tower ripped safety lines near the summit (Bowley, 2011).

In contrast, during recent years there have been increasing numbers of reports of climbers showing apparent disregard for the safety and suffering of sick climbers, and selfishness in the pursuit of mountaineering goals, including behaviors contributing to severe environmental degradation (Apollo, 2014, 2016). This suggests that traditional values are in some instances being subordinated by blind ambition, indifference to human suffering, and faulty leadership (Kodas, 2008). A widely reported controversial event on Everest (Sagamartha) in 2006 is an example. Over 40 ascending climbers, most with significant team back-up and radio contact to base camp, walked past a dying English mountaineer, David Sharp, as he sat collapsed and stranded 1 h from advanced high camp. Although climbing teams were well-equipped with modern equipment, oxygen and medicine, no significant medical assistance was given and no rescue attempts were made. Film footage of the unfortunate climber was taken, showing that David was able to speak despite his poor health status. All mountaineers walked around David and continued toward the summit (Breed and Gurubacharya, 2016)^1^. This led many to question whether some mountaineers place more value on a successful ascent of Everest than on the life of a fellow climber (Kodas, 2008).

It has also been reported that in 2004 a mountain guide, Gustavo Lisi, left his 69 year-old altitude-sick and delirious client, Dr. Nils Antezana, on the summit ridge of Everest. After descending to safety Lisi not only allegedly failed to raise the alarm about his client's plight but also posted news of his successful climb. Dozens of other climbers walked past the dying Dr. Antezana on their descent from the summit. The body of Dr. Antezana was lost to the mountain. It is presumed he fell to his death (Kodas, 2008). Pulitzer Prize–winning journalist Kodas has written a book with a disturbing number of similar accounts of “… a new breed of parasitic and predatory adventurer.”

Another extreme sport that is experiencing increasing morbidity and mortality is BASE jumping, which is arguably the most dangerous of the “extreme” sports (Mei-Dan et al., 2012, 2013). BASE jumping developed out of skydiving and uses specially adapted parachutes to jump from fixed objects. “BASE” is an acronym that stands for the four categories of fixed objects that one can jump off. These are: **B**uilding, **A**ntenna, **S**pan (a bridge, arch, or dome), and **E**arth (a cliff or other natural formation). It has been legally prohibited in many areas, most recently in Chamonix, France after a wingsuit BASE jumper crashed into a chalet potentially putting others at risk (Bisharat, 2017).

Monasterio and associates have examined the role of personality in extreme athletes with the Temperament and Character Inventory (TCI). The purpose of these studies has been to identify personality factors that may contribute to participation, accidents, and stress reactivity in mountaineers and BASE Jumpers (Mei-Dan et al., 2013; Monasterio et al., 2014, 2016).

The purpose of this paper is to hypothesize on the role of Self-Transcendence and its effect on individual personality profiles, in particular in moderating potentially self- destructive and regressive ethical, and moral behaviors in extreme sports. Beyond the research data presented, the proposed hypothesis is influenced by the experience of one of the author's (EM) who has extensive experience of “extreme sport” culture. EM has been involved in high-performance mountaineering, exploration and guiding for over 30 years. Reports of increasing death rates despite awareness of the risks among BASE jumpers are concerning. Furthermore, as indicated by the anecdotal examples already described, there are indications that in their pursuit of a summit some mountaineers can resort to unethical and at times criminal behaviors, whereas others adhere to the highest ethical standards and risk their life in the service of others.

## Self-Transcendence

Self-Transcendence (ST) is a trait associated with spiritual ideas and experiences, such as searching for something elevated and greater than one's individual self. ST is characterized by the direct perception of participation in something greater than one's self or perhaps even something boundless (Cloninger et al., 1993). Such a feeling of connectedness is a source of such joy and satisfaction that it can motivate people to act altruistically, even if that requires personal sacrifices and hardship, as exemplified by the honorable mountaineers already described here. Highly self-transcendent people have an outlook of unity and connectedness that motivates them to work in the service of others, instead of being preoccupied with individual accomplishments and self-aggrandizement (Cloninger et al., 1993).

## Cloninger's Temperament and Character Inventory (TCI)

**Figure 1:**
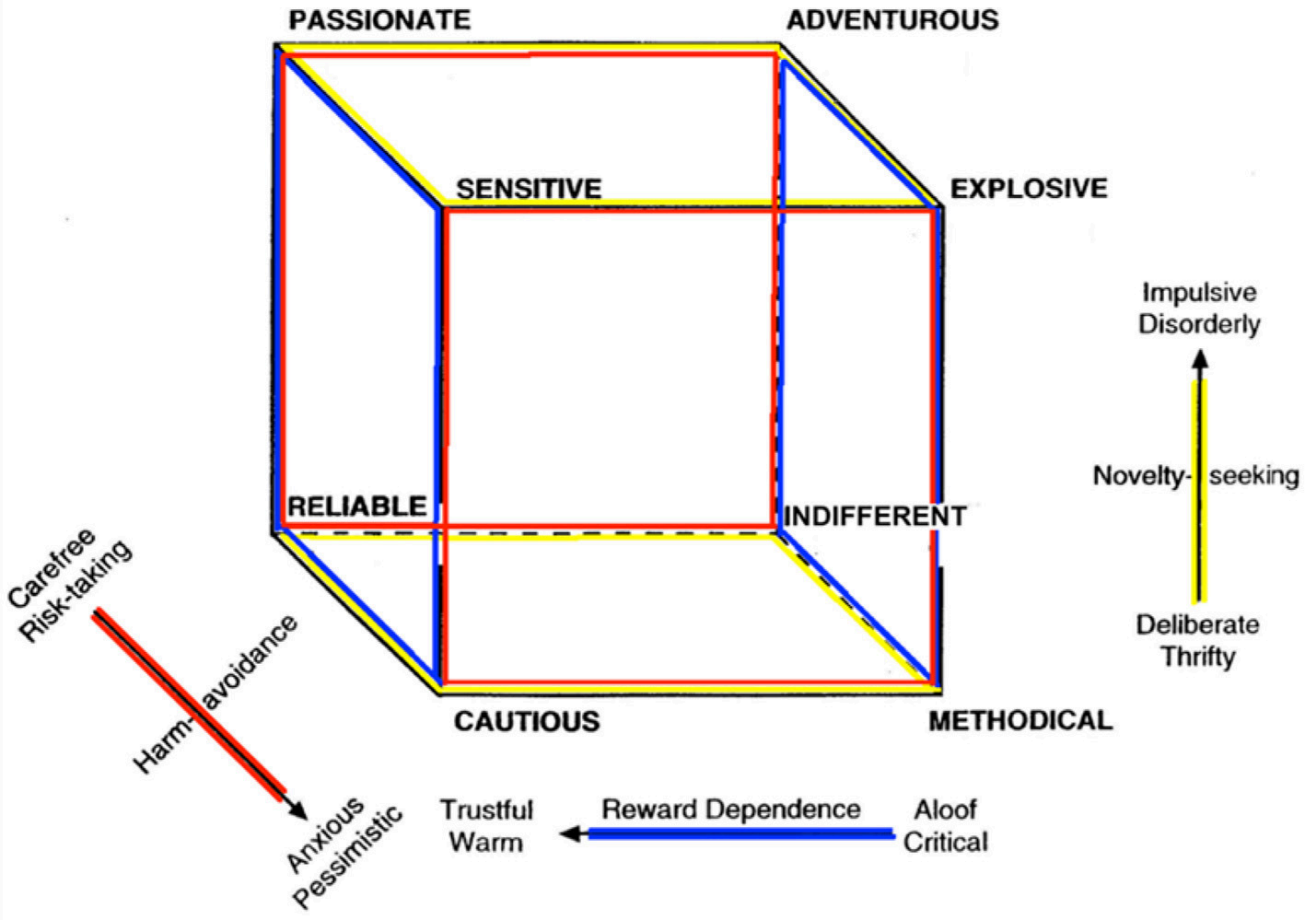
The Temperament Cube (Cloninger, 1987).

**Figure 2:**
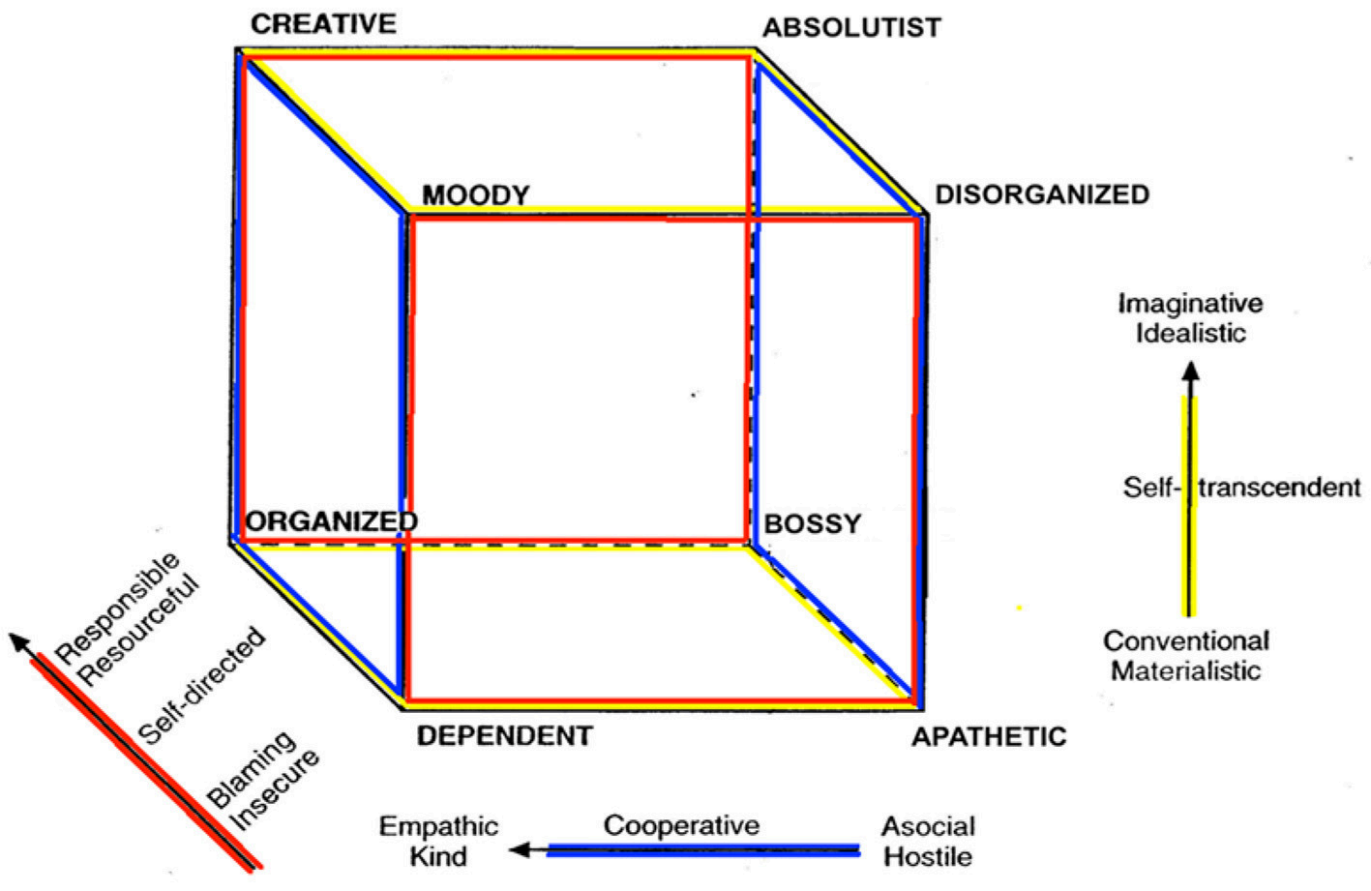
The Character Cube (Cloninger et al., 1993).

The TCI provides a comprehensive account of personality traits, measuring seven dimensions of personality (see Table 1) that are moderately heritable and associated with distinct brain networks and psychological characteristics (Cloninger, 1987; Cloninger et al., 1993; Gillespie et al., 2003; Van Schuerbeek et al., 2011). The model measures four dimensions of temperament (see Figure 1), which involve basic emotional drives modulated by the hypothalamus and related limbic structures (Lennox and Dolan, 2014), and three character dimensions (see Figure 2), which involve self-regulation of emotions and attention in order to achieve intentional goals and values regulated mainly in the neocortex (Cloninger, 1987; Cloninger et al., 1993; Gillespie et al., 2003; Van Schuerbeek et al., 2011). For example, high Self-Directedness is related to the executive attention system involving bipolar neurons in the anterior insula, frontal operculum, and anterior cingulate (Posner and Rothbart, 2007; Allman et al., 2011; Van Schuerbeek et al., 2011). Low Harm-Avoidance is associated with reduced functional connectivity in the insular salience network (i.e., right anterior insula with anterior cingulate and dorsolateral prefrontal cortex) (Paulus et al., 2003; Markett et al., 2013). Higher Novelty-Seeking and lower Harm-Avoidance are associated with larger volumes of cerebellar white matter and cortex bilaterally (Petrosini et al., 2015). Higher Novelty-Seeking scores are related to larger caudate and pallidum volumes bilaterally whereas lower Harm-Avoidance is related to reduced diffusivity in the putamen as measured by diffusion tensor imaging (Laricchiuta et al., 2014).

**Table 1:** Descriptors for high and low scorers on TCI subscales (Monasterio et al., 2016).

**TCI scales**	**TCI subscales**	**High scorers**	**Low scorers**
Novelty-seeking	NS1 excitability	Exploratory	Reserved
	NS2 impulsivity	Impulsive	Rigid
	NS2 extravagance	Extravagant	Thrifty
	NS4 disorderly	Rule-breaking	Orderly
Harm-avoidance	HA1 pessimism	Pessimistic	Optimistic
	HA2 fearfulness	Fearful	Risk-taking
	HA3 shyness	Shy	Outgoing
	HA4 fatigability	Fatigable	Vigorous
Reward- dependence	RD1 sentimentality	Sentimental	Objective
	RD2 sociability	Open	Secretive
	RD3 attachment	Friendly	Detached
	RD4 dependence	Approval-seeking	Independent
Persistence	PS1 eagerness	Enthusiastic	Hesitant
	PS2 hard-working	Determined	Easily discouraged
	PS3 ambition	Ambitious	Lazy
	PS4 perfectionism	Perfectionistic	Underachieving
Self-directedness	SD1 responsibility	Responsible	Blaming
	SD2 purposefulness	Purposeful	Aimless
	SD3 resourcefulness	Resourceful	Helpless
	SD4 self-acceptance	Hopeful	Hopeless
	SD5 self-actualizing	Self-actualizing	Unfulfilled
Cooperativeness	CO1 social tolerance	Tolerant	Prejudiced
	CO2 empathy	Empathetic	Self-centered
	CO3 helpfulness	Considerate	Hostile
	CO4 compassion	Forgiving	Revengeful
	CO5 conscience	Principled	Opportunistic
Self-transcendence	ST1 self-forgetfulness	Acquiescent	Controlling
	ST2 transpersonal identification	Altruistic	Individualistic
	ST3 spiritual acceptance	Spiritual	Skeptical

Extensive data on the reliability and validity of the TCI have been reported, and the TCI has been shown to have sound psychometric characteristics (Cloninger et al., 1993).

## Morbidity, Mortality, and TCI Results in Mountaineers and BASE Jumpers

The methodology and findings of these studies are reported in the literature (Monasterio, 2005; Mei-Dan et al., 2012, 2013; Monasterio et al., 2014, 2016). All participants volunteered their participation and were recruited from Alpine Club and BASE Jumpers group meetings and social media platforms, as well as personal communication among these sport communities. The mountaineers study specifically included only subjects who regularly engaged in climbing with a high level of technical proficiency. Ninety-five percent of mountaineers had more than 5 years climbing experience and 60% more than 10 years, with a median alpine grade of V+ (expert level) (Monasterio et al., 2014). BASE Jumpers had median participation rates of 6 years with >250 jumps (Mei-Dan et al., 2013; Monasterio et al., 2016).

The findings on average are consistent across all studies (refer to Table 2); these populations are adventurous in temperament with the “right stuff” (high Novelty-Seeking, low Harm-Avoidance, and Reward-Dependence) compared to low-risk sports participants and the general population. They are also highly self-controlled and organized in character (high in Self-Directedness and Cooperation, and low in Self-Transcendence). However, there is substantial heterogeneity in their individual personality profiles (Mei-Dan et al., 2013; Monasterio et al., 2014, 2016). Different configurations of temperament and character are illustrated in Tables 3, 4, respectively. These personality configurations vary in both their pattern of stress reactivity, resilience, and in their goals and values (Monasterio et al., 2016). Seventy five percent of the character configurations observed in BASE jumpers were associated with low Self-Transcendence (Monasterio et al., 2016). Likewise 85% of the character configurations in mountaineers were associated with low Self-Transcendence (Monasterio et al., 2014).

**Table 2:** Climber (*n* = 47), BASE jumper (*n* = 68), and Normative population (*n* = 181) TCI-235 score means (and SD).

	NS^**^	HA^***^	RD^**^	***P***	SD^**^	**C**	ST^***^
Climbers	21.36 (5.2)	9.1 (4.8)	14.1 (4.4)	5.0 (1.5)	35.5 (5.1)	34.1 (4.6)	11.2 (6.8)
BASE J.	22.8 (5.7)	7.9 (6.3)	13.8 (4.8)	5.5 (1.4)	33.4 (6.7)	33.7 (5.6)	12.7 (7.0)
Population	19.0 (5.8)	12.4 (6.9)	15.6 (4.3)	5.7 (2.1)	32.0 (7.0)	33.6 (6.7)	18.7 (6.3)

*Monasterio et al. (2012, 2014)*.

** p < 0.05;

*** p < 0.001. NS, Novelty Seeking;

*HA, Harm Avoidance; RD, Reward Dependence; P, Persistence; SD, Self Directedness; C, Cooperativeness; ST, Self Transcendence*.

**Table 3:** Distribution of BASE jumper temperament profile types (*n* = 98) (Monasterio et al., 2016).

**Profile type**	**Configuration**	**Number**	**Cumulative %**
Adventurous	Nhr	36	36.7
Passionate	NhR	18	55.1
Independent	nhr	14	69.4
Explosive	NHr	8	77.6
Methodical	nHr	8	85.7
Reliable	nhR	8	93.9
Cautious	nHR	4	98.0
Sensitive	NHR	2	100.0

**Table 4:** Distribution character configurations in Base jumpers (*n* = 98) (Monasterio et al., 2016).

**Profile type**	**Configuration**	**Number**	**Cumulative %**
Organized	SCt	43	43.9
Creative	SCT	18	62.2
Bossy	Sct	12	74.5
Apathetic	sct	10	84.7
Disorganized	scT	7	91.8
Dependent	sCt	4	95.9
Moody	sCT	3	99.0
Absolutist	ScT	1	100.0

## Discussion

The authors have utilized the TCI to identify important aspects of personality that contribute to participation and performance in expert level mountaineering and BASE Jumping. The TCI deconstructs personality into temperament and character dimensions that are composed of seven traits that vary widely in the general population. The range of configurations of these traits gives rise to a wide range of personality styles that are well-described in the literature (Cloninger, 1987). The study populations have adventurous personality profiles with organized character structure. The vast majority (85%) are considerably lower than average in Self-Transcendence, so we have focused on understanding the implications of this strong relationship in this article rather than the role of risk-taking that is more obvious. Extensive work on personality, risk-taking, executive attention, and brain circuitry have been extensively investigated (see earlier section: Cloninger's TCI).

*Temperament* refers to the automatic emotional responses or individual differences in the strength of drives underlying basic emotions that are moderately stable over time. The “adventurous” temperament profile, with high Novelty-Seeking and low Harm-Avoidance and Reward-Dependence, consistently identified in our study populations, is known to predispose not only to adventure seeking behaviors such as would be expected in a population of extreme athletes but also to antisocial behaviors. A number of studies have also identified the adventurous personality profile in populations of antisocial and psychopathic youth and adult criminal offenders (Cloninger et al., 1994; de Pádua Serafim et al., 2014; Lennox and Dolan, 2014). Therefore, temperament alone is not adequate to determine whether an individual person does or does not have a disordered personality and a higher risk of maladaptive behaviors.

The *character* dimensions can regulate emotional impulses and conflicts in such a way that a mature and healthy personality can develop regardless of the temperament. The healthiest personalities have consistently been found to be associated with high Self-Directedness and Cooperativeness. Conversely those with low scores in these traits consistently present with immaturity associated with disordered personalities (Cloninger et al., 1993). Antisocial and criminal behaviors in populations with the adventurous personality profile have been associated with low scores in all character traits of Self-directedness, Cooperativeness, and Self-Transcendence (de Pádua Serafim et al., 2014; Lennox and Dolan, 2014).

Among the relatively healthy personalities 2 character profiles are distinguished:

1. The “organized” character structure associated with high Self-Directedness and Cooperativeness, and low Self-Transcendence. People with organized characters are very responsible, organized, purposeful, and self-confident (high Self-Directedness). In addition they are tolerant, helpful and forgiving (high Cooperativeness). However, they are low in Self-Transcendence and so they are concerned with their own interests, and of those whom they regard as friends and associates with common goals and interests. As a result the organized character is very strong-willed, practical and goal oriented, driven mainly by achieving personal goals and ambitions and less influenced by altruism, idealism and spiritual concerns (Cloninger et al., 1994; Josefsson et al., 2013).
2. The “creative” character structure has high scores on all three character traits: they are high in Self-Directedness, Cooperativeness, and Self-Transcendence. Those with creative characters have the same capacity for resourceful productivity and helpful cooperation as those with organized characters, but they are also more intuitive and altruistic, and they strongly identify with nature, humanity, and with the universe as a spiritual whole. As such they are better able to tolerate uncertainty and ambiguity and are less egocentric in their outlook. Self-realization for individuals with the creative profile is determined by virtuous behaviors and values in the service to others and in seeking harmony with nature and the universe (Cloninger et al., 1994; Cloninger, 2013; Josefsson et al., 2013).

Taking the above into consideration we propose that the adventurous personality profile has a high activating tendency to engagement in risk-taking sports such as mountaineering and BASE Jumping. High Novelty-Seeking biases toward these exciting and challenging activities, with low Harm-Avoidance conferring confidence, vigor and low anxiety to deal with the inherent risks. Low Reward-Dependence is likely to contribute to independence and indifference to the opinion of others who may caution against engagement in these potentially dangerous sports. The potential antisocial tendencies associated with the adventurous personality under most circumstances are well-controlled by their organized character structure, which emphasizes disciplined initiative, clear goal-setting, collaboration and concern with maintenance of social norms.

However, we consider that the intense ambition to achieve a highly sought-after goal (mountain summit or a BASE jump) in the organized personality can in some situations become so dominant that it can lead to clouding of values with imprudence in self-care and care of others. It is likely that it also contributes to reckless exploitation of the mountain environment, as it is well-recognized that mountaineering is increasingly associated with environmental degradation and pollution (Apollo, 2014, 2016).

Highlighted in the introduction are examples of high-stake situations when mountaineers may have to forego the opportunity to reach a summit in order to assist or console an injured or disabled fellow climber. These situations demand critical decision-making under time constraints and often under duress from high altitude, cold, risk of avalanche and pressure from ambitious climbing partners. In these situations the ultimate desire and motivation to succeed on the ascent may, in some instances, supersede concern for the welfare of others, and erode traditional mountaineering values and acceptable human behavior, whereas in others it may lead to extraordinary acts of service in the benefit of others and self-sacrifice.

The personality profile of mountaineers is dominated by low Self-Transcendence. Individuals who score low on Self-Transcendence tend to be proud, impatient, self-preoccupied, and self-aggrandizing so that they often struggle to accept failure (Cloninger et al., 1994). Without the moderating effect of Self-Transcendence and its guidance toward altruism, the service of others and a sense of harmony with nature, the adventurous personality characteristic of mountaineers may in some (high-stake) situations manifest in considerable callousness, disregard and rationalization of controversial behaviors. High Self-Transcendence may contribute to the heroic decisions to act in the service of others instead of the pursuit of a summit.

BASE Jumping and its progression into wing-suit flying demands a very high level of performance. Wing suits act like wing parachutes and allow glide ratios that enable jumpers to glide away from cliffs and along canyons and ridgelines before deploying their BASE parachute (Mei-Dan et al., 2012). Given the speed of descent after take-off, achieving aerodynamic stability and determining a safe flight path requires a high level of performance under considerable stress. Sustained focus and meticulous attention to detail are imperative. The bias toward self-forgetfulness (i.e., absent-minded absorption) in search of transpersonal experiences that accompany Self-Transcendence is likely to be disadvantageous as this may lead to distractibility and diminished focus in jumpers. Therefore, low Self-Transcendence may be a key contributing factor to maintaining sustained focus and optimal decision-making in this population. However, the general tendency toward pride and seeking of fulfillment by succeeding in BASE jumping goals may contribute to more frequent engagement in the sport and the reported increased mortality (Mei-Dan et al., 2012).

Fortunately, both organized and creative characters can enhance their performance in extreme sports by training and practices that allow the development of both vigilance and altruism. The self-forgetfulness of the creative character involves a capacity for absorption in doing something that is valued, rather than being simply distractible. As a result, people with a creative character can learn to sustain the focus needed to look out for their safety along with the safety of others, which is a critical need in extreme sports (Rueda et al., 2011). Likewise people with organized character can develop greater altruism and joyful appreciation of nature through mindfulness of their connection with nature and other people (Campanella et al., 2014).

## Conclusion

Despite the substantial heterogeneity in their individual personality profiles mountaineers and BASE jumpers are adventurous in temperament and highly self-controlled and organized in character. The character configurations in these populations, mostly associated with low Self-Transcendence, may in some, critical situations manifest in considerable callousness, disregard and rationalization of controversial behaviors. More investigation is needed regarding training in mindfulness and attentional control as a standard component of preparation for extreme sports. Such training holds promise for enhancing the quality and value of the experience in a sustainable way that reduces risks.

## Ethics Statement

IRB approval from the University of North Carolina at Chapel Hill (IRB# 14-1942; approved 9/4/2014). This study was carried out in accordance with the recommendations of name of guidelines, name of committee with written informed consent from all subjects. All subjects gave written informed consent in accordance with the Declaration of Helsinki. The protocol was approved by the name of committee.

## Author Contributions

ME and CRC have been involved in the design of the studies, collection of data, interpretation of results and preparation of the manuscript.

### Conflict of Interest Statement

EM has previously worked as a mountain and jungle guide. The remaining author declares that the research was conducted in the absence of any commercial or financial relationships that could be construed as a potential conflict of interest.

## Footnotes

^1^Turkish climbers about David Sharp: “He was not part of a team.” Posted on explorers 479 web 5.6.2006 http://www.explorersweb.com/everest_k2/news.php?id=10049
